# Characterization of human iPSC-derived sensory neurons and their functional assessment using multi electrode array

**DOI:** 10.1038/s41598-024-55602-8

**Published:** 2024-03-12

**Authors:** Minami Hiranuma, Yuichi Okuda, Yuuka Fujii, Jean-Philippe Richard, Tomohisa Watanabe

**Affiliations:** 1 REPROCELL, Yokohama, Japan; 2 REPROCELL USA, Beltsville, MD USA

**Keywords:** Electrophysiology, High-throughput screening, Induced pluripotent stem cells, Sensory processing, Phenotypic screening

## Abstract

Sensory neurons are afferent neurons in sensory systems that convert stimuli and transmit information to the central nervous system as electrical signals. Primary afferent neurons that are affected by non-noxious and noxious stimuli are present in the dorsal root ganglia (DRG), and the DRG sensory neurons are used as an in vitro model of the nociceptive response. However, DRG derived from mouse or rat give a low yield of neurons, and they are difficult to culture. To help alleviate this problem, we characterized human induced pluripotent stem cell (hiPSC) derived sensory neurons. They can solve the problems of interspecies differences and supply stability. We investigated expressions of sensory neuron related proteins and genes, and drug responses by Multi-Electrode Array (MEA) to analyze the properties and functions of sensory neurons. They expressed nociceptor, mechanoreceptor and proprioceptor related genes and proteins. They constitute a heterogeneous population of their subclasses. We confirmed that they could respond to both noxious and non-noxious stimuli. We showed that histamine inhibitors reduced histamine-induced neuronal excitability. Furthermore, incubation with a ProTx-II and Nav1.7 inhibitor reduced the spontaneous neural activity in hiPSC-derived sensory neurons. Their responsiveness was different from each drug. We have demonstrated that hiPSC-derived sensory neurons combined with MEA are good candidates for drug discovery studies where DRG in vitro modeling is necessary.

## Introduction

Sensory neurons contribute to the transmission of a variety of intrinsic and environmental information such as temperature, touch, muscle length, pain, and itch. The cell bodies of sensory neurons are located in the dorsal root ganglia (DRG)^1,2^. DRG are primary afferent neurons induced by non-noxious and noxious stimuli. There are three main subtypes classified by specific function which are nociceptors, mechanoreceptors and proprioceptors^3,4^. Nociceptors respond to noxious heat and chemical stimuli and convey pain sensations. They are relatively small-to-medium diameter cells and classify into thin myelinated (Aδ) or unmyelinated (C) axons^1,5^. C-fibers respond to both peptidergic and non-peptidergic neurotransmitters^5^. Mechanoreceptors respond to touch sensations and temperature that is not considered noxious heat and cold. They are relatively large diameter cells and have thick myelinated (Aβ) axons^6,7^. Proprioceptors sense limb movement and position. They are large diameter cells^3^.

Recently, various differentiation protocols of human induced pluripotent stem cell (hiPSC) derived sensory neurons have been established^8–10^. Their expression levels of sensory neuron related genes and proteins are close to DRG, which are expected to be considered as research tools for drug discovery. Although, a few reports are investigating their function, most of them are patch-clamp and calcium imaging methods^8,9^.

Multi-Electrode Array (MEA) is readily used to research electrophysiological studies of electrically active cells. MEA can measure the field potential or activity across an entire cell network resting on electrodes, over time, with greater data points per well. Types of data that can be detected are superior than traditional assays such as patch-clamp electrophysiology that probe single neurons^11^. Some reports show electrophysiological drug responsiveness of DRG using MEA, however, it is difficult to culture and maintain spontaneous neural activity^12^. Furthermore, the often-used DRG derived from mouse or rat gives a low yield of neurons^13^. HiPSC-derived sensory neurons offer the advantage of convenient access and representing human physiology and pathology more closely than rodent models.

We report genes and proteins expression and also responsiveness of non-noxious and noxious stimuli using MEA to characterize the hiPSC-derived sensory neurons. We show their ability to be used for drug discovery as potent disease models of itching or pain, using histamine, a histamine inhibitor and a Nav1.7 inhibitor. These responses were similar to the ones reported for DRG. HiPSC-derived sensory neurons can be supplied in large quantities compared with the limited access we have to DRG. They are well-suited for a MEA characterization assay considering that more cells are needed for this type of assay when compared to patch-clamp and calcium imaging. Thus, we demonstrate that hiPSC-derived sensory neurons combined with MEA are strong candidates for in vitro modeling for drug screening and can be an alternative to DRG.

## Results

### Characterization of the expression of hiPSC-derived sensory neurons

**Figure 1:**
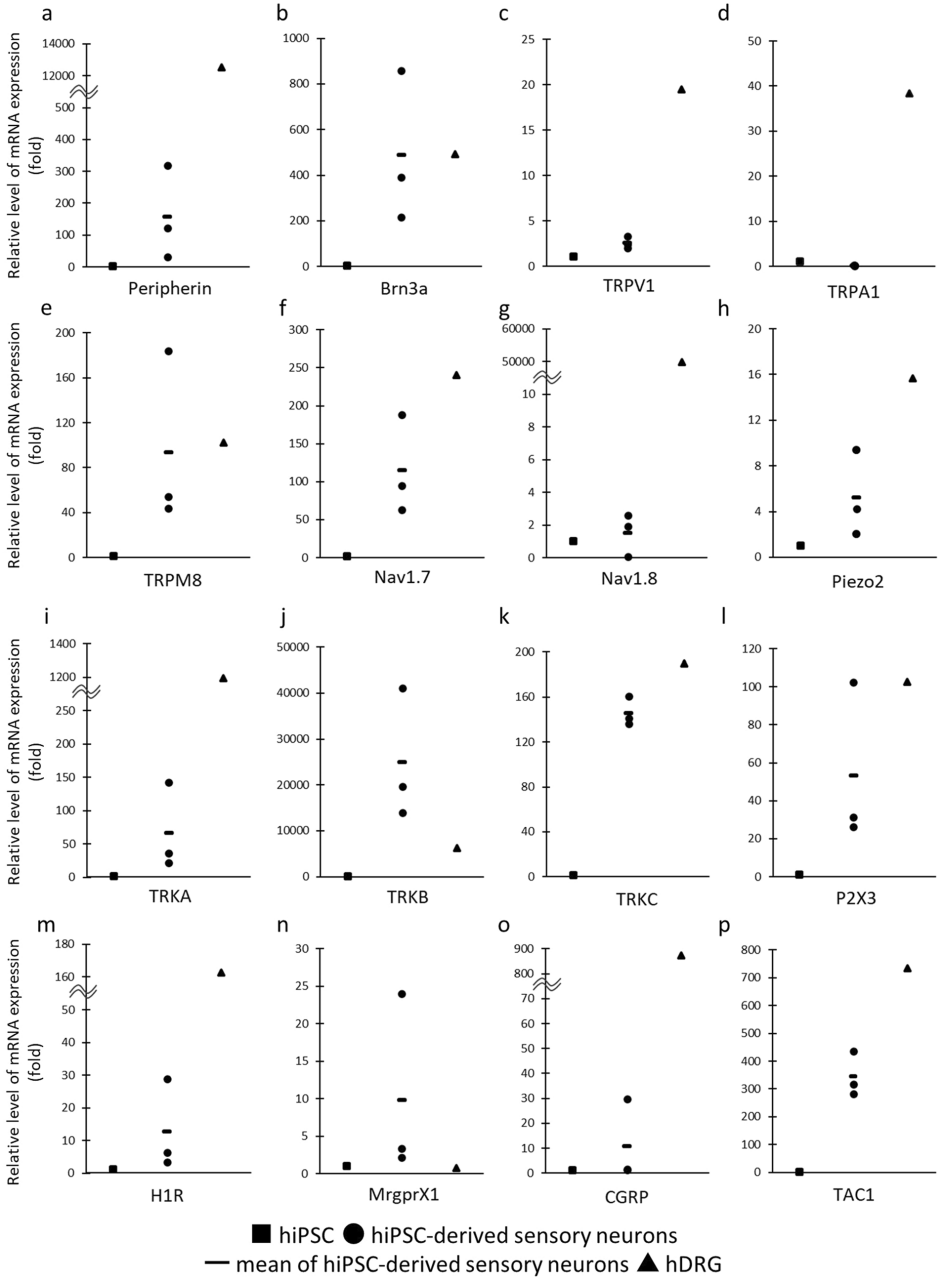
Expression of sensory neuron related genes in hiPSC, hiPSC-derived sensory neurons and human DRG. Real-time PCR showed expression of (**a**) Peripherin, (**b**) Brn3a, (**c**) TRPV1, (**d**) TRPA1, (**e**) TRPM8, (**f**) Nav1.7, (**g**) Nav1.8, (**h**) Piezo2, (**i**) TRKA, (**j**) TRKB, (**k**) TRKC, (**l**) P2X3, (**m**) H1R, (**n**) MrgprX1, (**o**) CGRP, (**p**)TAC1. The square marker, the circle marker and triangle marker indicate expression of genes in hiPSC, hiPSC-derived sensory neurons and human DRG respectively. Three different lot of hiPSC-derived sensory neurons were examined. The line marker represents the mean expression of genes in hiPSC-derived sensory neurons.

**Figure 2:**
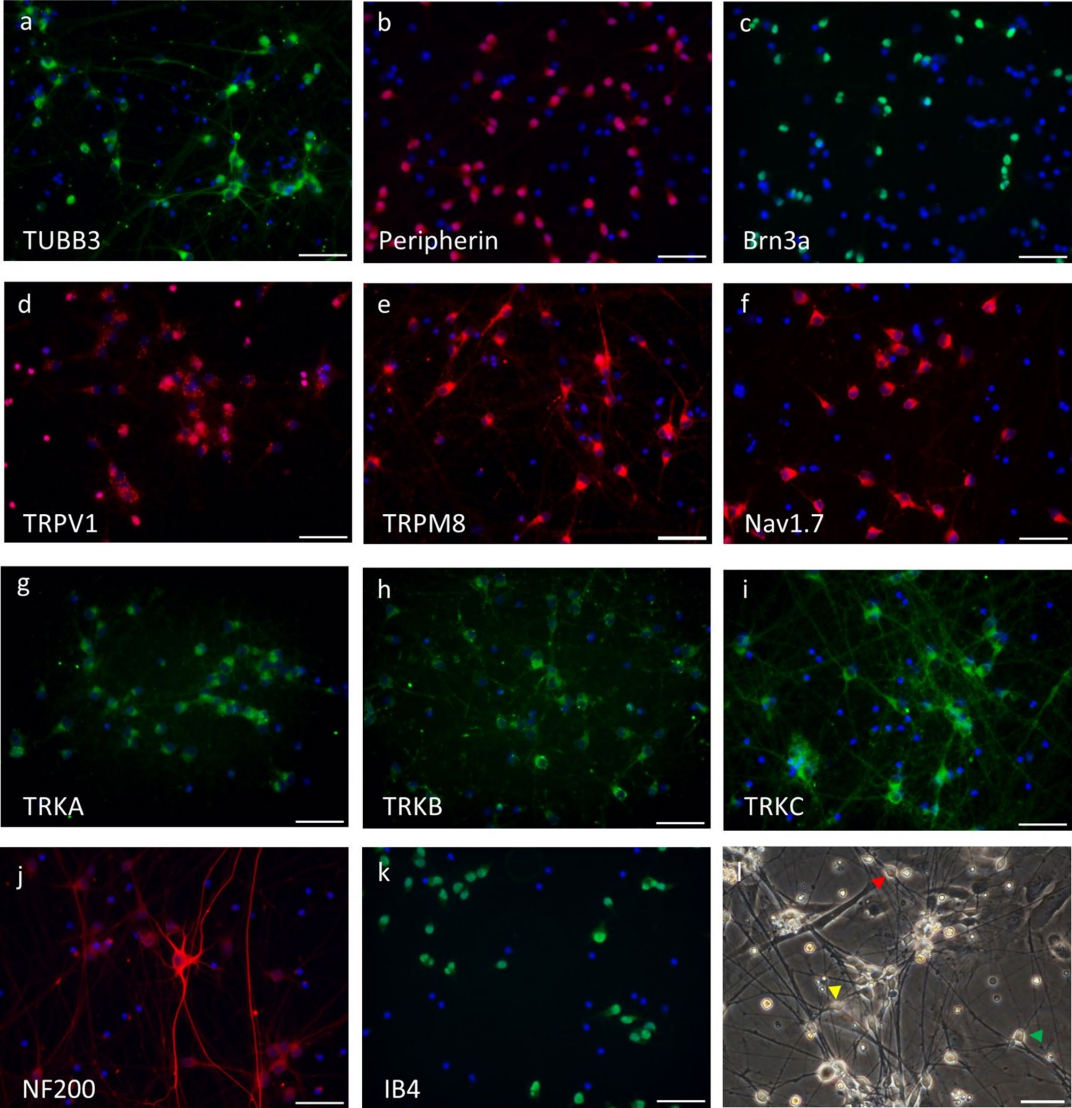
Expression of sensory neuron related proteins in hiPSC-derived sensory neurons and their morphology. The cells are stained for (**a**) TUBB3, (**b**) Peripherin, (**c**) Brn3a, (**d**) TRPV1, (**e**) TRPM8, (**f**) Nav1.7, (**g**) TRKA, (**h**) TRKB, (**i**) TRKC, (**j**) NF200, (**k**) IB4. DAPI stain of nuclei is shown in blue. (**l**) Image of iPSC-derived sensory neurons which exhibit a bipolar (red arrowhead), pseudounipolar (yellow arrowhead), or multipolar morphology (green arrowhead). Scale bar represents 50 µm.

HiPSC-derived sensory neurons (cat #RCDN004N, Reprocell.Inc) were used in this study. To identify characteristics of human iPSC-derived sensory neurons, we confirmed sensory neuron-related genes and proteins expression by real-time PCR and immunocytochemistry (ICC). Real-time PCR shows an increase in *Peripherin, Brn3a, TRPV1, TRPM8, Nav1.7, Nav1.8, Piezo2, TRKA, TRKC, TRKB, P2X3, H1R, MrgprX1, CGRP* and *TAC1* compared with hiPSC cultured 14 days in vitro (DIV) (Fig. 1). The nociceptor phenotype consists of Aδ-fibers, and C-fibers. C-fibers respond to both peptidergic and non-peptidergic neurotransmitters. HiPSC-derived sensory neurons expressed *TRKA* (nociceptor marker), IB4 (Aδ-fibers marker), *CGRP* and *TAC1* (peptidergic neurotransmitters) and *P2X3* (ATP (non-peptidergic neurotransmitter) receptor) (Figs. 1i,l,o,p and 2k). Furthermore, *TRPV1, TRPM8, Nav1.7* and *Nav1.8* which are nociceptors receptors were also expressed (Fig. 1c,e–g). TRPV1 is known to be activated by capsaicin and noxious heat (≥ 43 °C)^14^. TRPM8 is known to be activated by menthol, noxious (< 15 °C) and non-noxious (28–15 °C) heat^15,16^. Nav1.7 and Nav1.8 are known to be subtype of voltage-gated sodium channels which is preferentially expressed in nociceptors^17–19^. The mechanoreceptor phenotype consists of relatively large diameter cells that are Aβ-fibers. HiPSC-derived sensory neurons expressed *TRKB* (mechanoreceptor marker), NF200 (A-fibers marker), *TRPM8* and *Piezo2* (mechanoreceptor receptors) (Figs. 1e,h,j and 2j)^4^. *TRKC*, a proprioceptor marker was expressed in hiPSC-derived sensory neurons (Fig. 1k). Thus, these data suggests that the hiPSC-derived sensory neurons generated constitute a heterogeneous population of sensory neuronal subclasses. The expression of *TRPA1* in hiPSC-derived sensory neurons was lower than the one in hiPSC (Fig. 1d). The expression levels of *Brn3a, TRPM8, TRKB* and *MrgprX1* in hiPSC-derived sensory neurons were comparable to those in human DRG, whereas the others were lower than in hDRG. The reason some genes of hiPSC-derived sensory neurons showed lower expression than hDRG might be due to the immature nature of the hiPSC-derived sensory neurons^20^. Although we cultured them for a long time, the expression levels of *Peripherin, TRPV1, TRPA1, Nav1.7, Nav1.8, H1R,* and *CGRP* were not comparable to the ones in hDRG (Supplementary Fig. S1). Therefore, we confirmed proteins expression by ICC.

ICC showed expression of TUBB3 (mature neuron marker), Peripherin (peripheral neuron marker) and Brn3a (sensory neuron marker) at 14 DIV (Fig. 2a–c). TRPV1, TRPM8, Nav1.7, TRKA, TRKB and TRKC were expressed at the membrane (Fig. 2d–i). Since TRPV1, and TRPM8 are receptors of noxious and non-noxious stimulation and are expressed at the membrane, we expected them to be available for characterizing their function using MEA (Fig. 2d,e). NF200, a A-fibers marker, was expressed at a higher-intensity in relatively large diameter cells than small diameter cells (Fig. 2j). Although adult human DRG do not bind IB4 which is a non-peptidergic C-fibers marker, it was expressed in hiPSC-derived sensory neurons (Fig. 2k)^21^. The research showed expression of IB4 in prenatal human DRG at 8-month of gestation^22^. This data suggests that hiPSC-derived sensory neurons might be immature.

It is known that when observing rat DRG cells in the early stages of development, their morphology changes from bipolar cells to pseudounipolar cells^23^. Our hiPSC-derived sensory neurons exhibit a bipolar, pseudounipolar and multipolar morphology (Fig. 2l). A majority of the hiPSC-derived sensory neurons were bipolar neurons. This image suggests that our hiPSC-derived sensory neurons contained neurons with different degrees of maturity.

Taken together, hiPSC-derived sensory neurons express sensory neuron-related genes and proteins. They constitute a heterogeneous population of nociceptors, mechanoreceptors, and proprioceptors, and they differ in maturity. Thus, we proceeded to characterize their function next.

### Characterizing the response of hiPSC-derived sensory neurons to noxious and non-noxious stimulation

**Figure 3:**
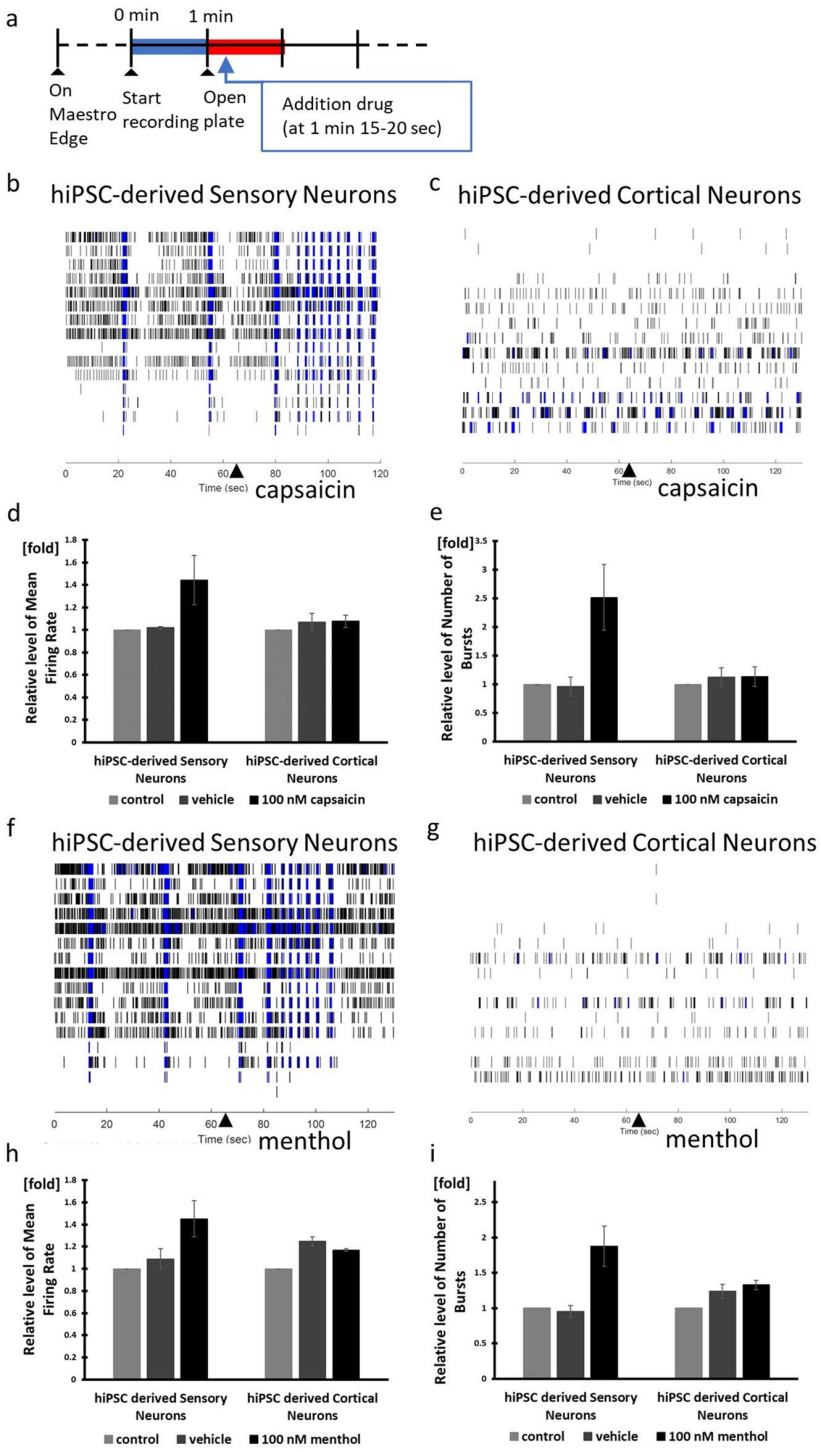
Capsaicin and menthol responsiveness using MEA. (**a**) Timeline of drug treatment. Baseline and dose response were recorded for 60 s when treating with capsaicin or menthol. Capsaicin experiment raster plots of (**b**) hiPSC-derived sensory neurons and (**c**) hiPSC-derived cortical neurons. The triangle marker indicates the time of capsaicin addition. (**d**, **h**) Mean Firing Rate normalized to the control. Control firing rate is calculated as firing rate before adding vehicle or drug. (**e**, **i**) Number of Bursts normalized to the control. Menthol experiment raster plot of (**f**) hiPSC-derived sensory neurons and (**g**) hiPSC-derived cortical neurons. n = 3 wells.

We confirmed whether hiPSC-derived sensory neurons responded to capsaicin, menthol, noxious heat (43–46 °C), which are noxious stimuli, and bradykinin, and non-noxious heat (37–42 °C), which is a non-noxious stimulus^14–16,24^_._ Sensory neurons of DRG are used as in vitro model of nociceptive response. DRG responded to 10 nM, 100 nM, 1 µM capsaicin^25,26^. We measured and compared data before and after drug treatment (Fig. 3a). We measured the response to treatment with 100 nM capsaicin which resulted in increase in Mean Firing Rate (MFR) and Number of Bursts (NOB), whereas vehicle treatment had no effect on them (Fig. 3d,e). Capsaicin-evoked activity is known to be rapid in DRG^25^. This result showed that neural activity was evoked within 10 s after treatment with capsaicin in hiPSC-derived sensory neurons as well (Fig. 3b). To determine whether capsaicin-evoked neuronal activity is characteristic of hiPSC-derived sensory neuron, we treated with 100 nM capsaicin in hiPSC-derived cortical neurons. As a result, hiPSC-derived cortical neurons did not respond to capsaicin (Fig. 3c–e). Moreover, we added 100 nM AMG9810 which is a TRPV1 antagonist for 60 min before treating with capsaicin. A response to capsaicin was not observed in the presence of AMG9810 (Fig. S2a,b). These data suggest that capsaicin-evoked activity occurred via TRPV1 in iPSC-derived sensory neurons. Thus, we can conclude that hiPSC-derived sensory neurons specifically respond to noxious stimulus and could be used in functional assays using MEA.

Menthol activates TRPM8 which is a nociceptive receptor. Since mouse and rat DRG respond to 10 µM and 100 µM menthol, we decided to treat with the same concentrations^26,27^. The high concentration of menthol resulted in suppressing spontaneous neural activity (Supplementary Fig. S3). This may be due in part to the higher expression of TRPM8 in hiPSC-derived sensory neurons than in human DRG (Fig. 1e, Supplementary Fig. S1e). Treatment with 100 nM menthol resulted in an increase in MFR and NOB in hiPSC-derived sensory neurons whereas hiPSC-derived cortical neurons did not respond to menthol (Fig. 3f–i). The data show that menthol got a response from nociceptive-like and non-nociceptive-like DRG neurons^28^. Since our hiPSC-derived sensory neurons responded to menthol, they may also include functionally non-nociceptive like neurons.

**Figure 4:**
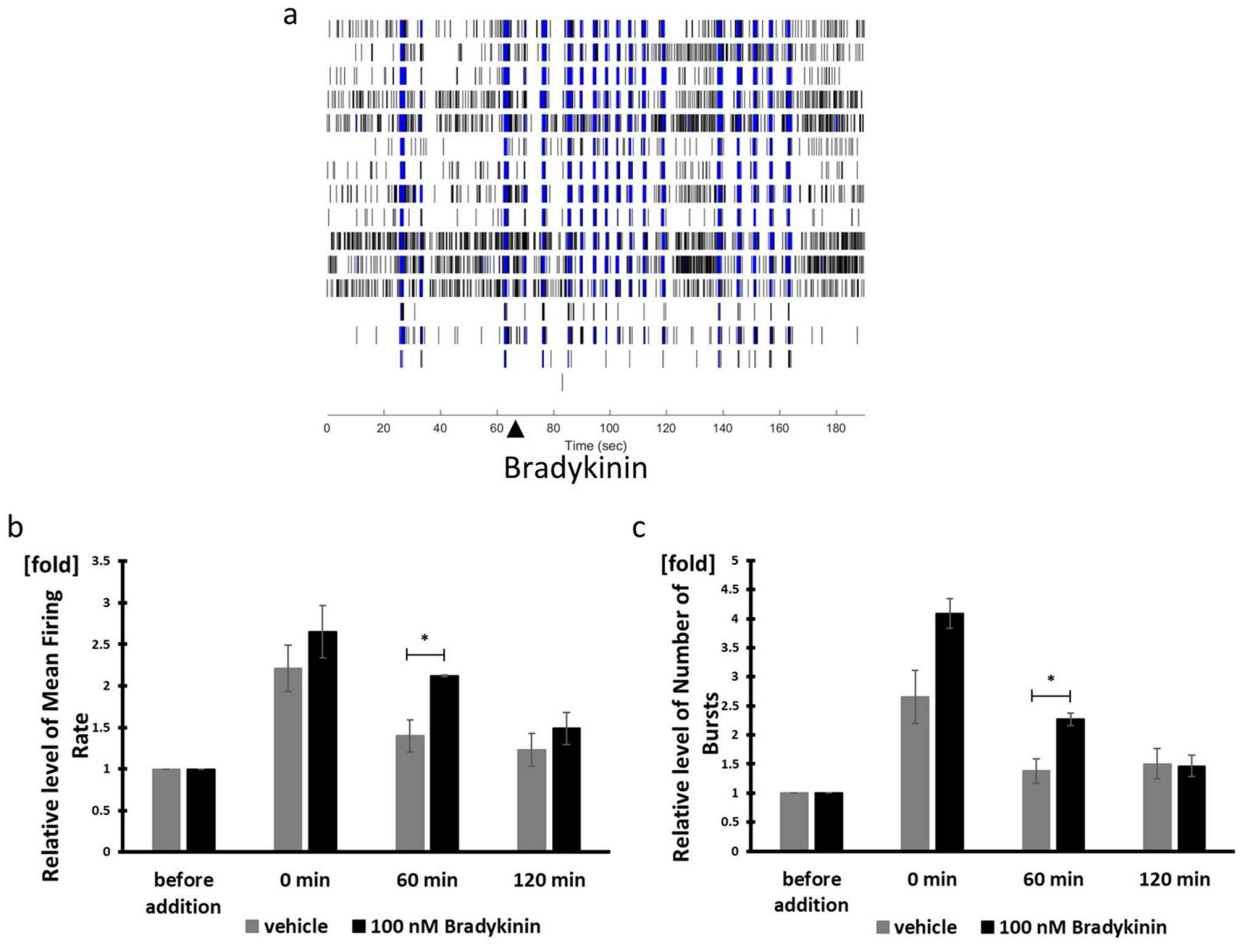
Bradykinin responsiveness. (**a**) Bradykinin experiment raster plot of hiPSC-derived sensory neurons. The triangle marker indicates the time of bradykinin addition. (**b**) Mean Firing Rate after addition of Bradykinin normalized to firing rate before addition of Bradykinin. (**c**) Number of Bursts after addition of Bradykinin normalized to number of bursts before addition of Bradykinin. n = 3 wells, *p < 0.05.

Bradykinin activates nociceptors and causes pain^,^^24^. Treatment with 100 nM bradykinin resulted in significant increase in MFR and NOB compared to vehicle treatment for 60 s (n = 3, *p* < 0.05) (Fig. 4a–c). In contrast to capsaicin and menthol, the onset of bradykinin-evoked neural activity was relatively long (Figs. 3b,f and 4a). Bradykinin-evoked activity increased gradually and reached its mean peak at 60 s in DRG^25^. However, hiPSC-derived sensory neurons were able to respond faster than DRG, because they also responded to an additional stimulation which immediately activated them when bradykinin and vehicle were added against the well of the MEA plate. There is expression of TRKB and Piezo2 relevant to touch sensation in hiPSC-derived sensory neurons, explaining why they may have responded to an additional stimulation.

**Figure 5:**
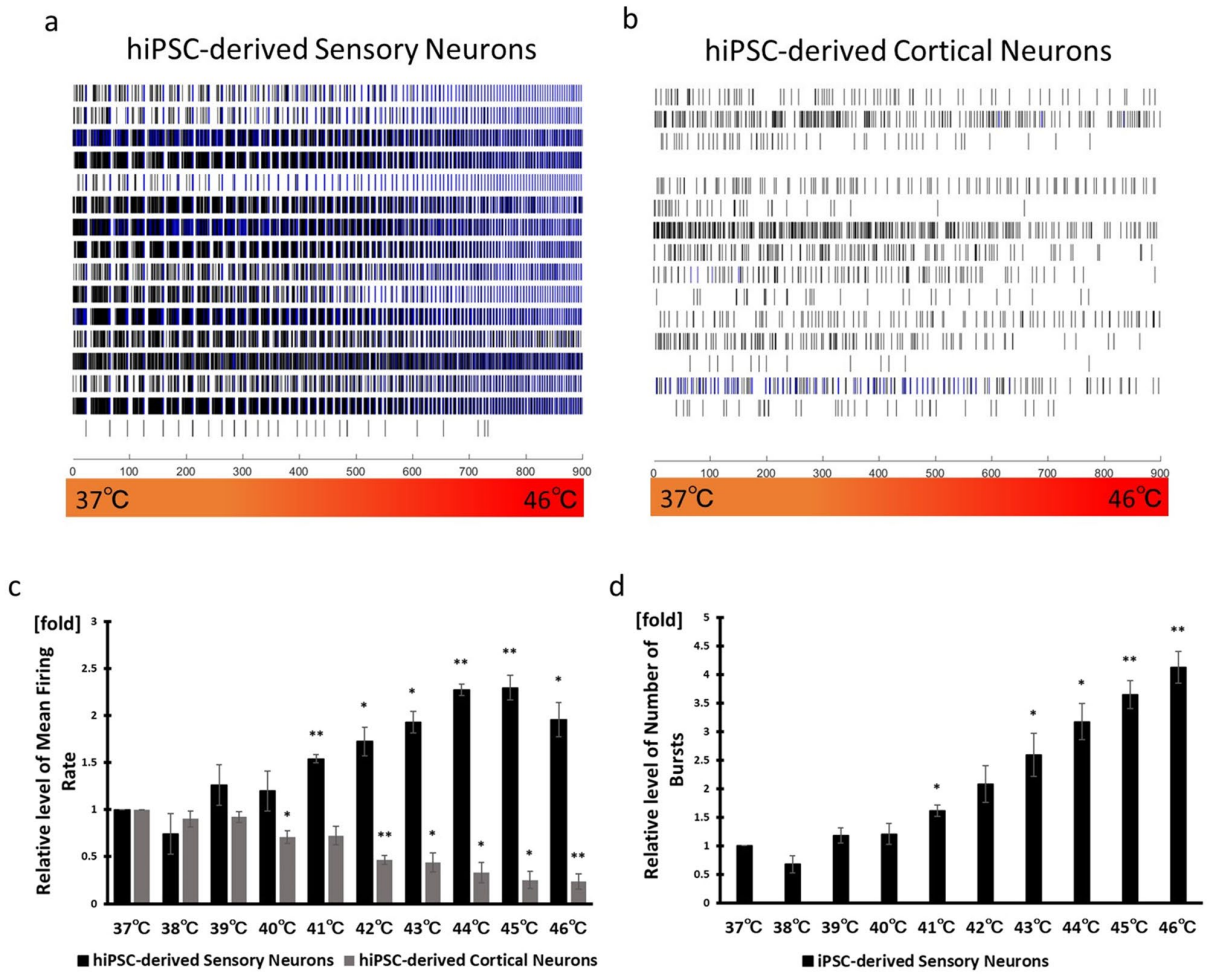
Temperature responsiveness. Raster plots of (**a**) hiPSC-derived sensory neurons and (**b**) hiPSC-derived cortical neurons when the temperature is gradually increased from 37 to 46 °C. (**c**) Mean Firing Rate normalized to the firing rate at 37 °C. (**d**) Number of Bursts normalized to the number of bursts at 37 °C. The data for the number of bursts in hiPSC-derived cortical neurons isn’t shown because one of the three wells didn’t produce any burst. n = 3 wells, *p < 0.05, **p < 0.01 compared with the corresponding value at 37 °C. Functional assessment of hiPSC-derived sensory neurons against itching stimuli

MFR were observed to increase gradually in DRG, when temperature increases from 37 to 42 °C via the stage plate heater, part of the recording system^25^. We increased the temperature from 37 to 46 °C via MAESTRO’s system to confirm responsiveness to noxious heat and non-noxious heat. We observed that MFR and NOB increased gradually and reached their mean peak at 45 °C and 46 °C respectively, in hiPSC-derived sensory neurons (Fig. 5). In the presence of TRPV1 antagonist, AMG9810, MFR were lower than that of vehicle at 43–46 °C (Fig. S2c,d). Because TRPV1 is known to be activated by noxious heat (≥ 43 °C), these results suggest that TRPV1 may contribute to the response to 43–46 °C in iPSC-derived sensory neurons. The relative levels of MFR (1.54 ± 0.046) and NOB (1.62 ± 0.01) at 41 °C, which is non-noxious heat, were significantly higher than those at 37 °C. However, MFR decreased gradually in hiPSC-derived cortical neurons with an increase in temperature (Fig. 5b,c).

These data suggest that the observed and recorded response is specific to sensory neurons and the hiPSC-derived sensory neuron populations generated in this study are likely to include nociceptors that respond to noxious stimuli like capsaicin, menthol, bradykinin, and noxious heat (≥ 43 °C) and to include mechanoreceptors that respond to non-noxious stimuli (41 °C).

### Itch responsiveness of hiPSC-derived sensory neurons

Atopic dermatitis (AD) is the most common chronic skin disease which causes a global disease burden^29^. AD causes itch (pruritus) and poor non-health-based quality-of-life. It is known that itch occurs via C-fiber in nociceptors^30^. Recently, investigating itch has been established by using human sensory neurons from stem and other progenitor cells as in vitro model^31^. Although substances causing itch treat to their cells, their inhibitors effect aren’t confirmed using hiPSC-derived sensory neurons. Because we demonstrated that our hiPSC-derived sensory neurons expressed nociceptor genes and proteins, and responded to noxious stimuli, we expected that they also responded to an itch stimulus and its inhibitor.

**Figure 6:**
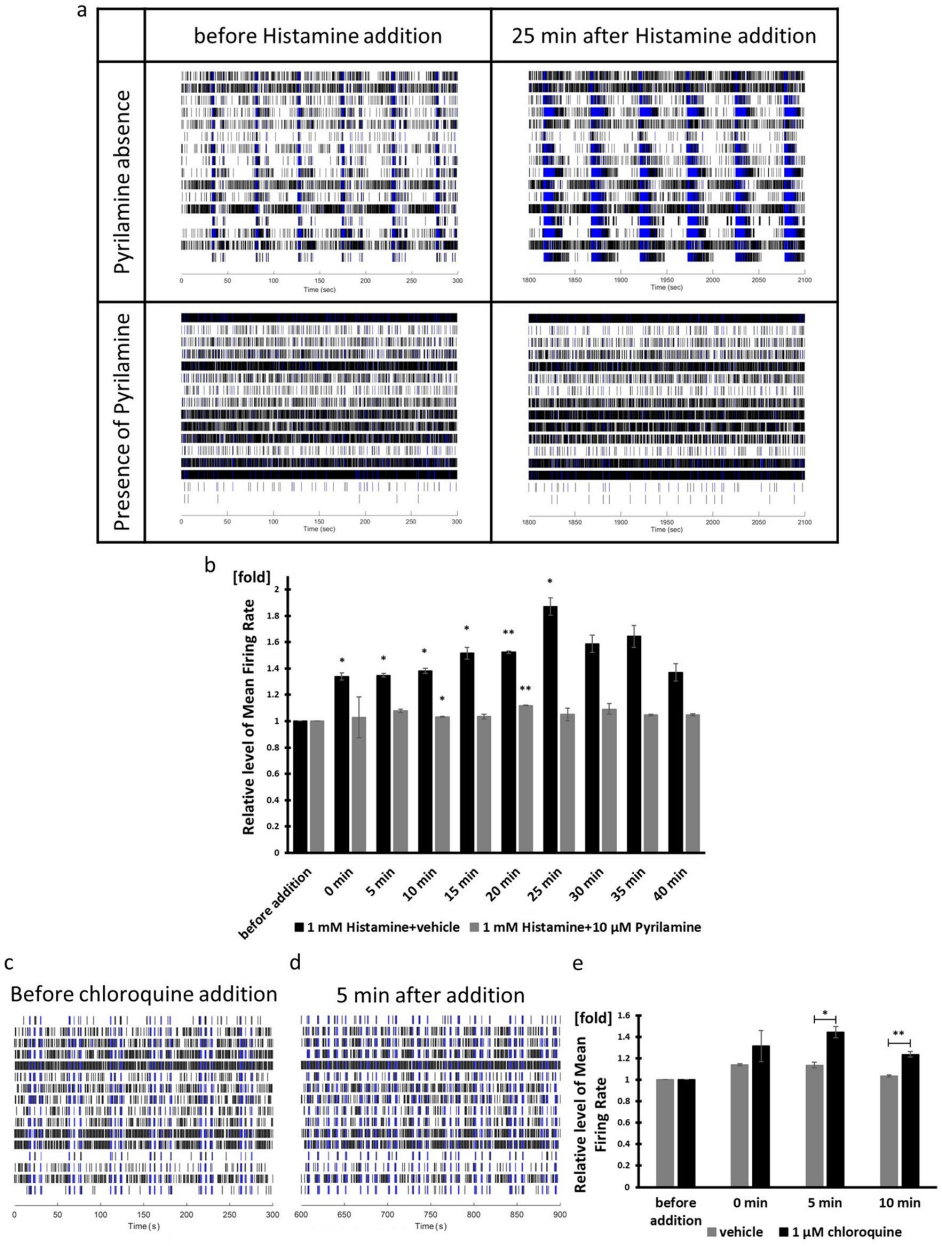
Histamine, H1R inhibitor, pyrilamine and chloroquine responsiveness. (**a**) The left raster plots have been recorded before histamine addition. The right raster plots have been recorded 25 min after histamine addition. Upper raster plots are recorded in pyrilamine absence. Lower raster plots are recorded with presence of pyrilamine. (**b**, **e**) Mean Firing Rate normalized to firing rate before drug addition. Raster plots of (**c**) before chloroquine addition and (**d**) 5 min after chloroquine addition. The experiment with histamine and pyrilamine was performed with n = 2 wells each. Experiment with chloroquine was performed with n = 3 wells. *p < 0.05, **p < 0.01 compared with the value recorded before addition or with vehicle.

Histamine is one of the substances that cause itching via C-fiber. The histamine receptor is a four G protein-coupled receptor. Histamine H1 receptor (H1R) is involved in the induction of histamine-induced pruritus^32^. Since we confirmed that the H1R gene is expressed, we examined whether hiPSC-derived sensory neurons respond to histamine, using MEA. Mouse DRG responded to 100 µM Histamine, as described in the literature^33^. HiPSC-derived sensory neurons didn’t respond to 100 µM Histamine but responded to 1 mM Histamine (Supplementary Fig. S3d–f and Fig. 6a,b). The MFR gradually increased and reached its mean peak at 25 min. Pyrilamine is a histamine H1 receptor inverse agonist. We treated the sensory neuron population with 10 µM Pyrilamine for 60 min before adding Histamine. As a result, Pyrilamine inhibited Histamine-evoked activity (Fig. 6a,b). These results suggest that Histamine-evoked activity occurred via H1R in iPSC-derived sensory neurons.

Chloroquine is a drug that has been used in the treatment to prevent malaria. Histamine-independent pruritus is known to be one of the side effects of chloroquine^34^. Mrgprs are receptors of chloroquine and are activated by it^35^. Since we confirmed expression of human MrgprX1 by real-time PCR, we investigated the potential response of hiPSC-derived sensory neurons by chloroquine. DRG are reported to respond to 1 mM chloroquine, however the MFR gradually decreased at the same concentration in hiPSC-derived sensory neurons (Supplementary Fig. S3g,h)^36^. 1 µM chloroquine increased the MFR and reached the mean peak after 5 min of incubation (Fig. 6c–e). The mean peak after stimulation with chloroquine was reached faster than after stimulation with histamine.

These data showed an example of the effect of an itch inhibitor and different responses between itch inducing drugs. HiPSC-derived sensory neurons may be available for drug discovery against AD.

### Inhibitor responsiveness of Nav1.7 channels involved in pain

**Figure 7:**
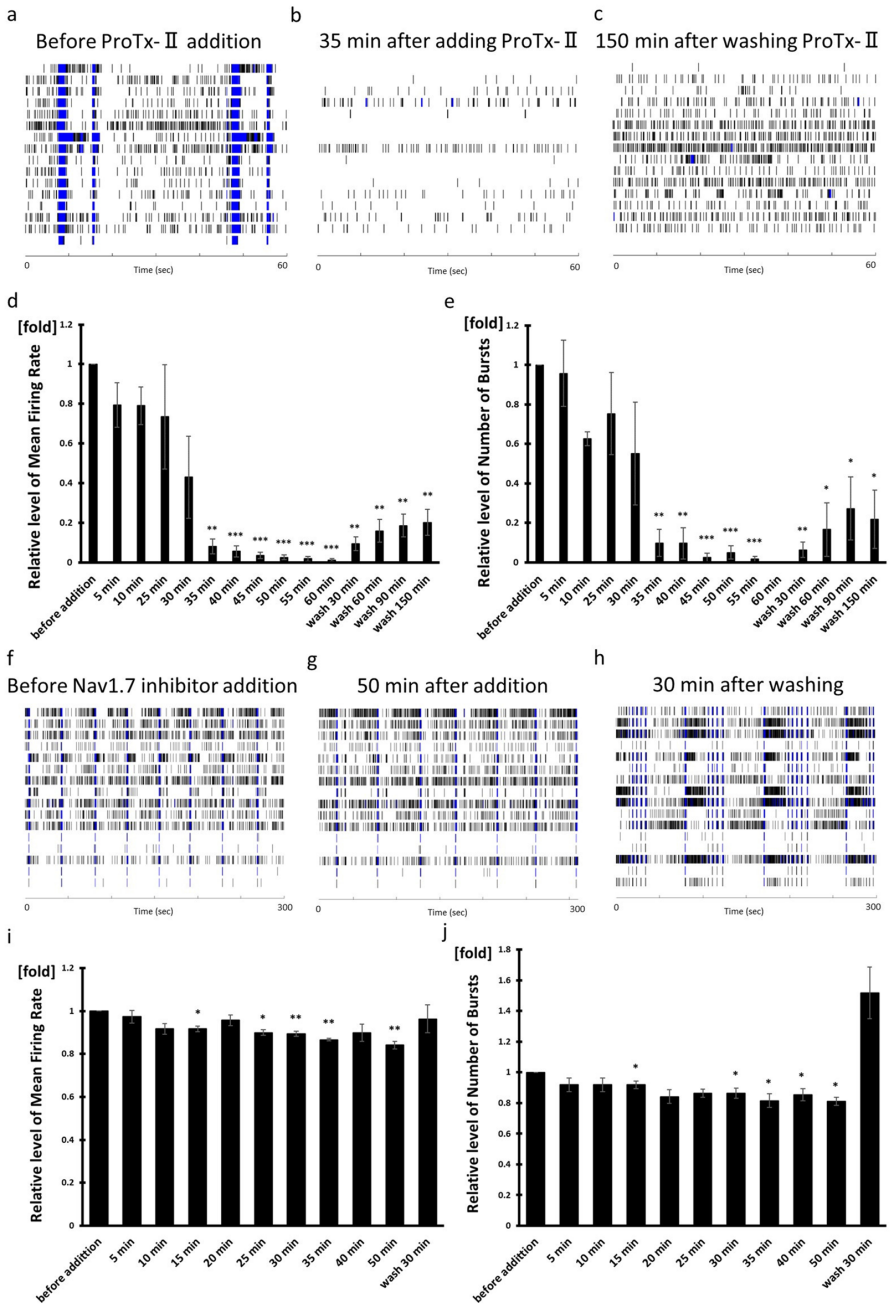
Nav1.7 channels inhibitor, ProTx-II and Nav1.7 inhibitor responsiveness. Raster plots of (**a**) before ProTx-II addition (baseline), (**b**) 35 min after adding ProTx-II and (**c**) 150 min after washing ProTx-II, respectively. (**d**, **i**) Mean Firing Rate and (**e**, **j**) Number of Bursts normalized to mean firing rate and number of bursts before drug addition. Raster plots of (**f**) before Nav1.7 inhibitor addition (baseline), (**b**) 50 min after adding Nav1.7 inhibitor and (**c**) 30 min after washing Nav1.7 inhibitor, respectively. n = 3 wells, *p < 0.05, **p < 0.01, ***p < 0.001 compared to the value recorded before drug addition.

Nav1.7 subtype of voltage-gated sodium channels is expressed in DRG. Mutations in the gene encoding Nav1.7 are associated with either absence of pain or with exacerbation of pain. Recently, Nav1.7 has been an attractive target to pursue treating pain. ProTx-II is a tarantula venom peptide that preferentially inhibits Nav1.7 over other Nav subtypes^37^. It suppressed spontaneous neural activity in a time dependent manner in hiPSC-derived sensory neurons (Fig. 7a–e). The MFR and NOB are significantly diminished after 35 min of incubation with 1 µM ProTx-II (Fig. 7d,e). After washing out ProTx-II, the suppressed neural activity gradually recovered. Although, responses was completely blocked by 300 nM ProTx-II in rodent DRG, the responses in hiPSC-derived sensory neurons were not blocked at the same concentration^38^. This may be due in part to the lower expression of Nav1.7 in hiPSC-derived sensory neurons than in human DRG.

ProTx-II is known to act not only as a Nav1.7 inhibitor but also to act on Nav1.5 channels and on some T-Type Ca^2+^ channels^39,40^. Thus we administered a small molecule inhibitor that is more selective for Nav1.7^41^. Although 300 nM of a more selective Nav1.7 inhibitor suppressed MFR and NOB in a time dependent manner in hiPSC-derived sensory neurons, the degree of decrease was lower than that of ProTx-II (Fig. 7f–j). After washing it out, the suppression of the neural activity was lifted.

These results suggested that hiPSC-derived sensory neurons may serve as a drug screening tool for pain.

## Discussion

We confirmed the expression of nociceptor, mechanoreceptor and proprioceptor related genes and proteins. We found that the hiPSC-derived sensory neurons generated in this study constitute a heterogeneous population of sensory neuronal subclasses. HiPSC-derived sensory neurons also responded to noxious and non-noxious stimuli using MEA in the same way as DRG. Furthermore, we confirmed that they responded to electrical stimulation (Supplementary Fig. S4a–d). We showed the use of hiPSC-derived sensory neurons with histamine and Nav1.7 inhibitors. We have demonstrated that hiPSC-derived sensory neurons combined with MEA are a powerful tool for drug discovery and can substitute DRG as in vitro models.

Although most of the genes in hiPSC-derived sensory neurons were higher than the ones in hiPSC, it is difficult to approach the expression levels in DRG even after maturation the sensory neuronal networks for 128 DIV (Supplementary Fig. S1). Previous reviews have reported that some gene expression levels in iPSC-derived peripheral sensory neurons were lower than those of hDRG^20,31^. Full maturation of hiPSC derived neurons has not yet been achieved and remains a goal in the field. However, critical proteins in our hiPSC-derived sensory neurons were expressed at the membrane. Furthermore, their function and responses as sensory neurons were similar as DRG. For example, in the case of capsaicin and temperature treatment, it is known that CGRP and substance P are released from DRG when TRPV1 is activated by capsaicin or heat stimulation^42,43^. CGRP and substance P enhance neuron excitability in DRG^44,45^. HiPSC-derived sensory neurons express TRPV1, CGRP and substance P (splicing variant of TAC1) and respond to capsaicin or heat stimulation. Thus, our study suggests that hiPSC-derived sensory neurons can be used in functional assays even if their gene expression levels are not comparable to those of DRG.

MEA is well-suited for characterizing neural models because it can easily measure the field potential of an entire cell network being cultured on electrodes. Some studies have reported that rodent DRG can be used as in vitro nociceptor models for phenotype assay using MEA^12,25^. However, rodent disease modeling can fail to replicate human biology and pathology. Also, human donor limitation explains the scarcity of human DRG use. Human iPSC-derived sensory neurons could solve these problems. Although human sensory neurons from stem and other progenitor cells have to be kept for long times in culture to be used in functional assays due to the immaturity of their protein expression and electrophysiological network, our results show that the MFR observed after 4 DIV, exceeded the one observed for DRG after 14 DIV, and peaked above 10 Hz after 35 DIV (Supplementary Fig. S4e)^12,25,31^. HiPSC-derived sensory neurons can reliably be used for recording until 64 DIV. The functional assays in this report were performed between 27 and 61 DIV and some of the same wells of neuronal cultures were used again after washing out the previous drug.

It is notable that the formation of a network among the hiPSC sensory neurons does not occur in DRG. We treated the cultures with synaptic blockers, NMDA receptor antagonist PEAQX and AMPA receptor antagonist NBQX, to confirm whether they can reduce the spontaneous firing. As a result, the combination of PEAQX and NBQX reduced MFR, NOB and Number of Network Bursts (NONB) the most (Fig. S5). Furthermore, the degree of reduction of NOB was higher than that of MFR and NONB. Thus, we can conclude that our hiPSC-derived sensory neurons include non-sensory neurons that form synaptic connections and propagate excitability throughout the neural network.

In vivo, under physiological conditions, DRG neurons do not display spontaneous activity but only initiate action potentials when triggered by specialized sensors (noci-, mechano-, proprioceptors). DRG neurons are generally silent in most studies without stimulations in vitro. The research showed NGF and IL-6 activated them^12,25^. We considered that our hiPSC-derived sensory neurons exhibited spontaneous firing because sensory neuron culture medium used in this study contains neurotrophins and also spontaneous non-sensory neurons. Although spontaneous neural activation exists in hiPSC-derived sensory neurons, they responded to bradykinin and histamine which trigger inflammatory responses. These responses are similar to DRG responses activated by inflammatory factors. Taken together, the hiPSC-derived sensory neurons generated in this article could be used for characterizing neural model using MEA. These methods, when combined with optical imaging methods that utilize calcium dyes, could be powerful tools for high-throughput screening in the drug discovery field.

In vitro models of itching will help develop medical treatment for AD. We established that itch can be investigated using hiPSC-derived sensory neurons as in vitro model. Patient-derived sensory neurons could be generated by using their iPSC and these neurons could be useful for drug discovery of AD. Our results regarding the decreased histamine-induced neuronal excitability with pyrilamine treatment prove the potential of hiPSC-derived sensory neurons using MEA for drug discovery. Furthermore, our results show that their responsiveness to histamine and chloroquine differed which could potentially be used in assays for various types of itching. However, some drugs didn’t respond at the same concentrations as with DRG. It is likely attributable to different expression levels between hiPSC-derived sensory neurons and human DRG. A recent study showed that raster plots machine learning using hiPSC-derived cortical neurons predicted the seizure probability and assessed the concentration-dependent seizure probability of the drugs^46^. Thus, further studies on hiPSC-derived sensory neurons also need to be undertaken to establish methodologies for quantitative, as well as qualitative evaluations.

In summary, we described that hiPSC-derived sensory neurons could be used for drug discovery screening where disease modeling involves itching and pain. In addition, they can avoid interspecies difference, are easily available, and exhibit specific functions of sensory neurons. We propose that hiPSC-derived sensory neurons can be an alternative model to primary DRG.

## Material and methods

### Thawing and culture of hiPSC-derived sensory neurons

All experiments with hiPSC-derived sensory neurons were performed with Reprocell.Inc's hiPSC-derived sensory neurons (cat #RCDN004N, Reprocell.Inc). HiPSC-sensory neurons were differentiated from the StemRNA Human iPSCs line 771-3G (RCRP003N, Reprocell.Inc) and we followed the differentiation method referenced as Young et al.^8^. The expression pattern of sensory neuron-related genes showed features similar to those in the referenced paper. One vial of frozen sensory neurons was placed in a 37 °C-water bath and warmed for about 4 min, until completely thawed. Using a P1000 pipette, the cell suspension was gently added to a 50 mL sterile conical tube. 9 mL wash medium was added dropwise into this tube. The conical tube was centrifuged at 300 × g for 5 min at room temperature. When using a 24 well plate coated with iMatrix-511 silk (Nippi), the sensory neurons were seeded at 1.5 × 10^5^ cells per well at a density of 3 × 10^5^ cells/mL. When using a CytoView MEA 24 (AXION BIOSYSTEMS) plate coated with iMatrix-511 silk, sensory neurons were centrifuged and seeded at 1.5 × 10^5^ cells/5 µL per spot at a density of 30 × 10^6^ cells/mL. They were maintained in Sensory Neuron Culture Medium (cat#RCDN103, Reprocell.Inc) at 37 °C, 5% CO_2_. Half medium change was performed every 3–4 days.

### Thawing and culture of hiPSC-derived cortical neurons

All experiments in hiPSC-derived cortical neurons were performed with purchased line RCDN001N, (Reprocell.Inc). One vial of frozen neurons was placed in a 37 °C-water bath and warmed for about 90 s and decanted into wash medium and centrifuged and resuspended with Reproneuro Culture Medium (cat#RCDN101, Reprocell.Inc). When using a CytoView MEA 24 plate coated with iMatrix-511 silk, neurons were seeded at 2.0 × 10^5^ cells/5 µL per spot at a density of 40 × 10^6^ cells/mL. They were maintained at 37 °C, 5% CO_2_ and half medium changes were performed every 3–4 days.

### RNA extraction and quantitative real-time PCR analysis

Total RNA of hiPSC-derived sensory neurons and StemRNA Human iPSCs (771-3G) cultured with StemFit^®^AK02N (Ajinomoto) were extracted from cultured cell pellets using RNeasy Mini Kits (Qiagen), according to the manufacturer’s instructions. Total RNA of human dorsal root ganglion was purchased from Clontech, which is pooled from 21 male/female Caucasian individuals (ages: 16–65; cause of death: sudden death). RNA was used to synthesize cDNA using PrimeScript™ RT Master Mix (TaKaRa), according to the manufacture’s instruction. Real-time PCR was performed on Thermal Cycler Dice^®^ Real Time System III (TaKaRa) using TB Green^®^ Fast qPCR Mix (TaKaRa). The primers were listed in Supplemental Table 1. ΔCT values were obtained by normalizing to the mean of Actin-β. ΔΔCT values were obtained by normalization to ΔCT values of hiPSC samples. Relative expressions were used in the 2-ΔΔCT method.

### Immunocytochemistry

hiPSC-derived sensory neurons were fixed with 4% paraformaldehyde for 10 min, washed twice with 0.1% Bovine Serum Albumin (BSA) in PBS, permeabilized with 0.2% Triton X-100 for 10 min and then blocked in 1% BSA for 1 h at room temperature. Fixed samples were incubated with primary antibodies against TUBB3 (1:1000, Covance, PRB-435P), Brn3a (1:25, Millipore, MAB1585), Peripherin (1:200, Millipore, AB1530), TRPV1 (1:100, Invitrogen, PA1-748), TRPM8 (1:100, NOVUS, NBP1-97311), Nav1.7 (1:100, NOVUS, NBP2-12904), TRKA (1:80, R&D SYSTEMS, MAB1751R), TRKB (1:80, R&D SYSTEMS, MAB3971), TRKC (1:100, R&D SYSTEMS, AF373), Neurofilament 200 (1:1000, Sigma, N4142), Isolectin B4 (1:1000, Thermo Fisher Scientific, I21411) over night at 4 °C. The following day, three washes were performed in 0.1% BSA in PBS, and the cells were incubated with appropriate secondary antibodies (1:500, Invitrogen) for 1 h at room temperature. All samples were incubated with DAPI (Sigma). A BZ-X810 microscope (Keyence) was used for recording the fluorescence images.

### MEA analysis

Extracellular potentials were recorded using a MaestroEdge (AXION BIOSYSTEMS). The cells were cultured in a CytoView MEA 24 plate coated with iMatrix-511 silk for electrophysiological recording. Drug treatment recordings were performed between 27 and 61 days after thawing and seeding. Continuous channel data recording was performed at a sampling rate of 12.5 kHz/channel and filtered using a Butterworth band-pass filter (200–3000 Hz). The number of bursts were detected by Inter-Spike Interval Threshold (≤ 100 ms). All baselines were recorded after equilibrating at least 10 min on the MaestroEdge. For treatment with capsaicin, menthol, bradykinin and ProTx-II, baselines and post-drug recordings were performed for 60 s. For treatment with histamine, pyrilamine, chloroquine, Nav1.7 inhibitor, PEAQX and NBQX baselines and post-drug recordings were performed for 300 s. All vehicle treatments were measured for the same amount of time as the drug treatment before the drug treatment. Drugs were added carefully against the wall of the well with 5 µL/well. Due to the high concentration of histamine, 200 µL/well of the medium was removed once and added to the final concentration. The wells were washed two times with medium after recording and used for the next recording after at least 3 days. The heating plate experiment was performed via Axis Navigator software. The electrical stimulation was set at 300 Hz biphasic square pulse train (× 40 times) with an amplitude of 1.2 V and pulse duration of 750 µs/phase using the Axis Navigator software. Recording data were saved as a raw data file and outputted as an AxIS Spike and a CSV file using the Axis Navigator software. Raster plots were outputted from AxIS Spike files using the Neural Metric Tool software.

### Compound preparation

Capsaicin (Wako), menthol (Wako), bradykinin (Wako), ProTx-II (Wako), histamine (Wako), pyrilamine (Sigma), chloroquine (Wako), Nav1.7 inhibitor (GLPBIO), AMG9810 (Wako), PEAQX (GLPBIO) and NBQX (MedChemExpress) were dissolved in DMSO, ethanol or sterile, distilled water at the maximum concentration guaranteed by the manufacturer. The working concentration was prepared as 100 × dissolved in sterile, distilled water.

### Data analysis

Data are presented with standard error of mean (SEM). Statistical comparisons were made with an unpaired Student’s t test and paired t test and differences were considered significant at *p* < 0.05, 0.01, 0.001.

### Supplementary Information

Supplementary Information.

## Data Availability

The datasets used and/or analyzed during the current study available from M.H on reasonable request.
