# Supplementary material for: Characterization of human iPSC-derived sensory neurons and their functional assessment using multi electrode array
Source: Sci Rep. 2024 Mar 12;14:6011. doi: 10.1038/s41598-024-55602-8 (PMC10933446; doi:10.1038/s41598-024-55602-8)
Supplement: Supplementary file 1 — Supplementary Information. [file 41598_2024_55602_MOESM1_ESM.pdf]

Characterization of Human iPSC-derived Sensory Neurons and Their Functional Assessment Using Multi Electrode Array

Minami Hiranuma[1] *, Yuichi Okuda[1], Yuuka Fujii[1], Jean-Philippe Richard[2], Tomohisa Watanabe[1]
[1]REPROCELL, Yokohama, Japan
[2]REPROCELL USA, Beltsville, MD, USA

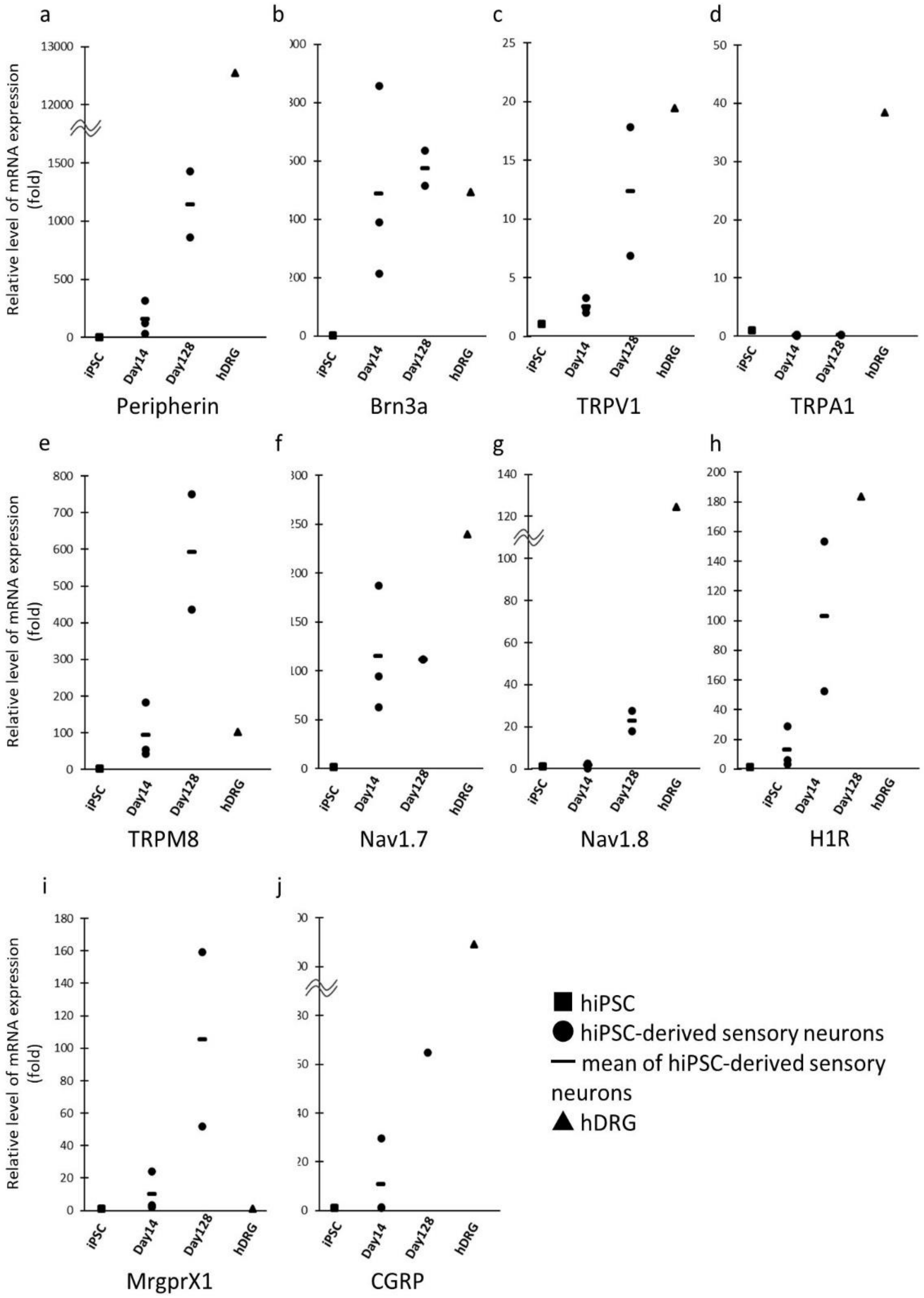

Supplementary FigureS1. Comparison of expression levels of sensory neuron-related genes in hiPSC-derived sensory neurons 14 days and 128 days after thawing and seeding. Real-time PCR shows expression of (a) Peripherin, (b) Brn3a, (c) TRPV1, (d) TRPA1, (e) TRPM8, (f) Nav1.7, (g) Nav1.8, (h) H1R, (i) MrgprX1, and (j) CGRP. The square marker, the circle marker and triangle marker represent expression of genes in hiPSC, hiPSC-derived sensory neurons and human DRG, respectively. The line marker represents the mean expression of

genes in hiPSC-derived sensory neurons. The sample size of day14 neurons and day128 neurons is three and two, respectively, with the exception of CGRP at day128 (n=1).

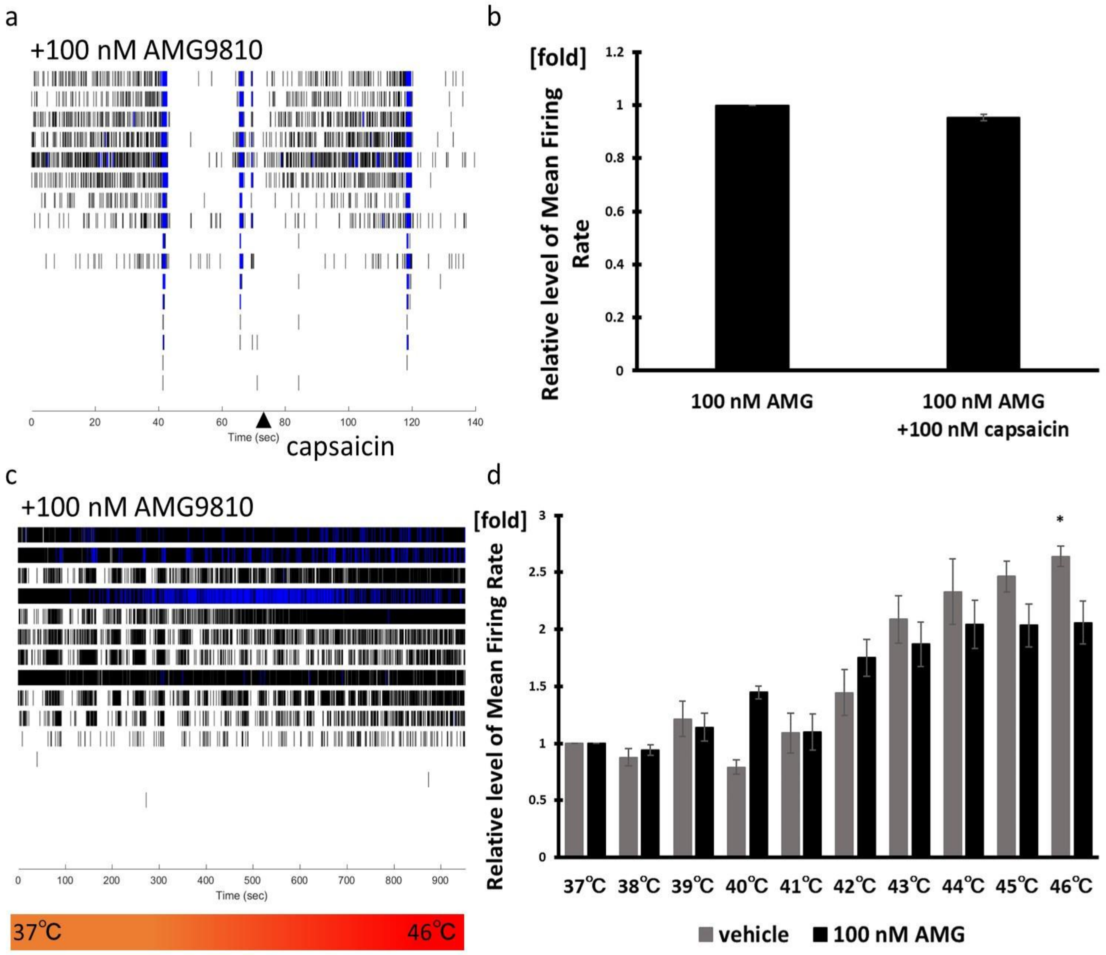

Supplementary FigureS2. Capsaicin and temperature responsiveness in the presence of TRPV1 antagonist, AMG9810. (a) Raster plots of iPSC-derived sensory neurons recorded for the 100 nM capsaicin treatment experiment in the presence of 100 nM AMG9810. The triangle marker represents the time of capsaicin addition. (b) Mean Firing Rate normalized to the Mean Firing Rate recorded before adding the drug (n=3 wells). Raster plots of (c) hiPSC-derived sensory neurons when the temperature is gradually increased from 37°C to 46°C in the presence of vehicle and 100 nM AMG9810. (d) Mean Firing Rate normalized to the firing rate at 37°C. (n=2 wells). *$p<0.05$ compared with the corresponding value at 37°C.

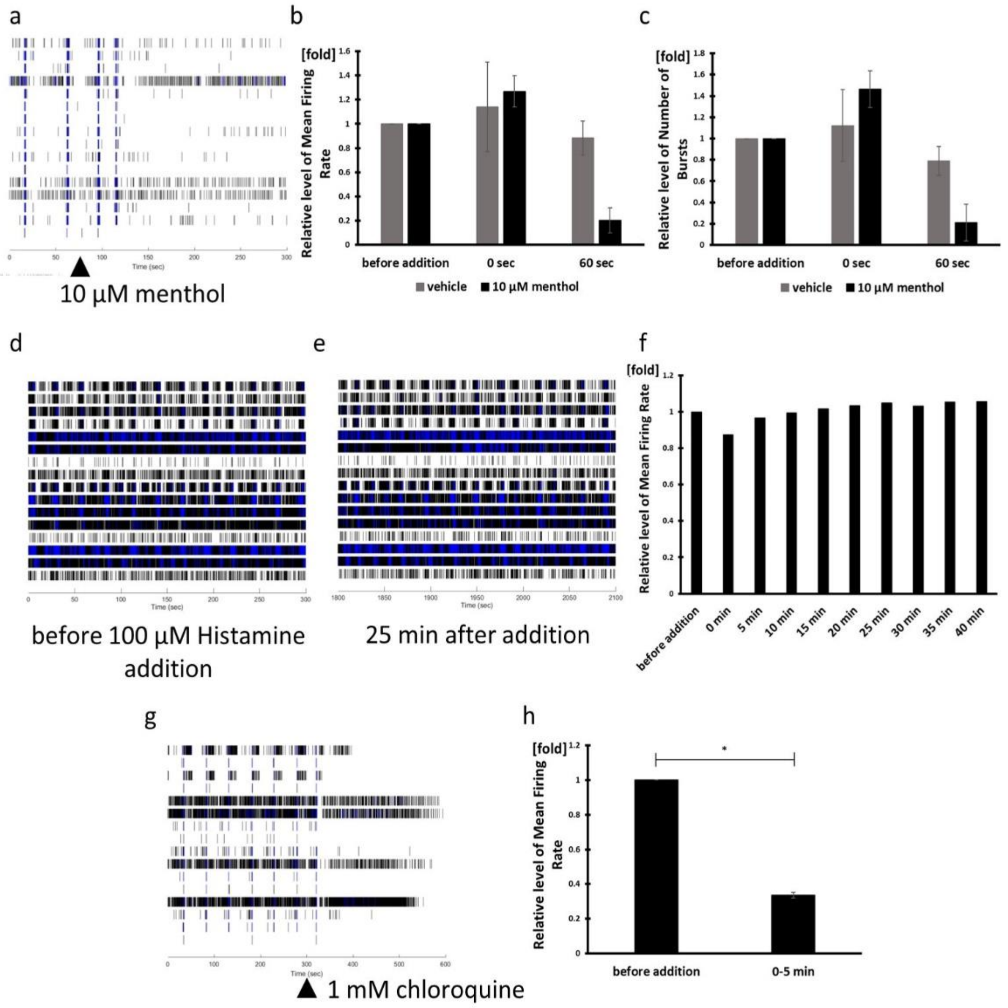

Supplementary FigureS3. Additional data for menthol, histamine and chloroquine treatment. (a) Raster plots of iPSC-derived sensory neurons recorded for the 10 μM menthol treatment experiment. The triangle marker represents the time of menthol addition. (b) Mean Firing Rate and (c) Number of Bursts normalized to the Mean Firing Rate and Number of Bursts recorded before adding the drug (n=3 wells). Raster plot (d) before the addition of 100 μM histamine and (e) 25 min after adding histamine. (f) Mean Firing Rate normalized to the Mean Firing Rate recorded before adding the drug (n=1 well). (g) Raster plots of iPSC-derived sensory neurons recorded during the 1 mM chloroquine treatment. (h) Mean Firing Rate normalized to the Mean Firing Rate recorded before adding the drug (n=2 wells). *p<0.05.

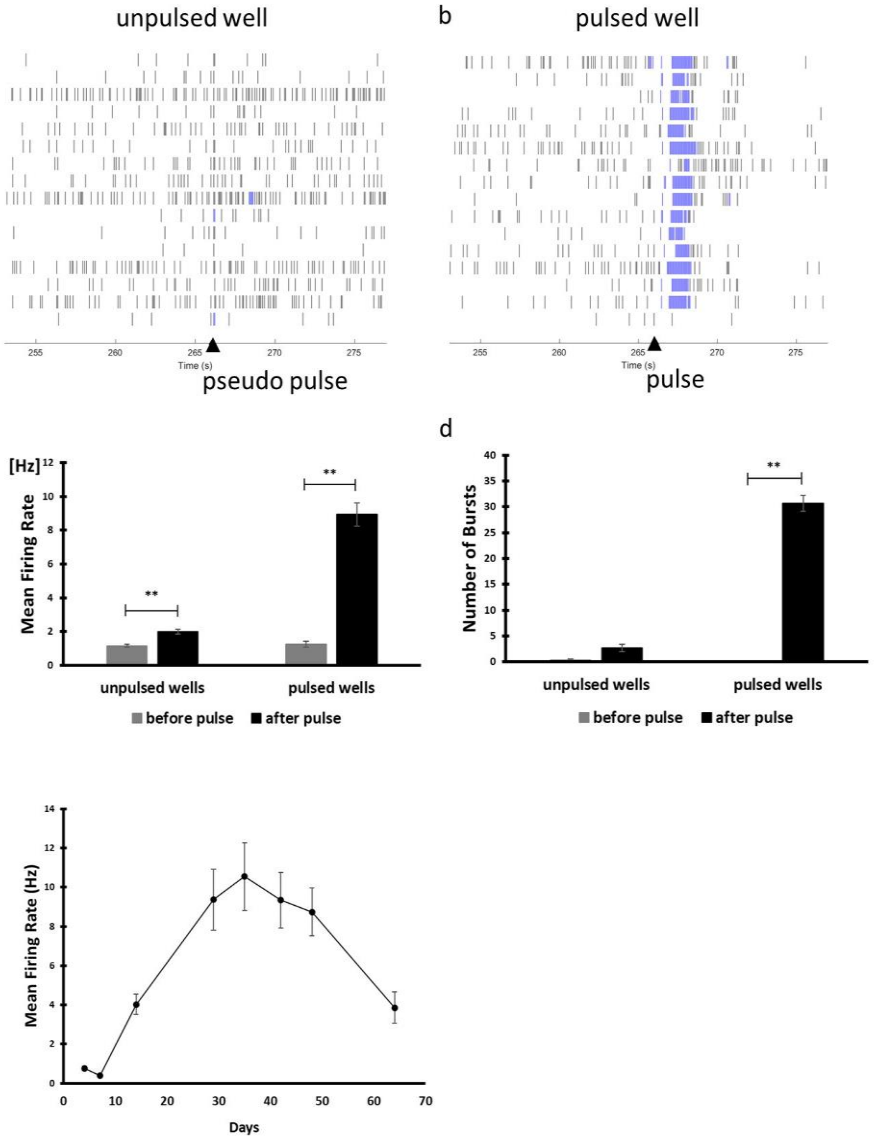

Supplementary FigureS4. Additional data on MEA analysis. Raster plots of (a)a unpulsed well shows no stimulation and (b) a pulsed well shows stimulation. The triangle marker represents the point of electrical stimulation. (c) Mean Firing Rate. (d) Number of Bursts. Analysis was performed on an interval of 12 seconds before and after the pulse. n=3 wells, **$p<0.01$. (e) Temporal change of MFR (n=5 wells).

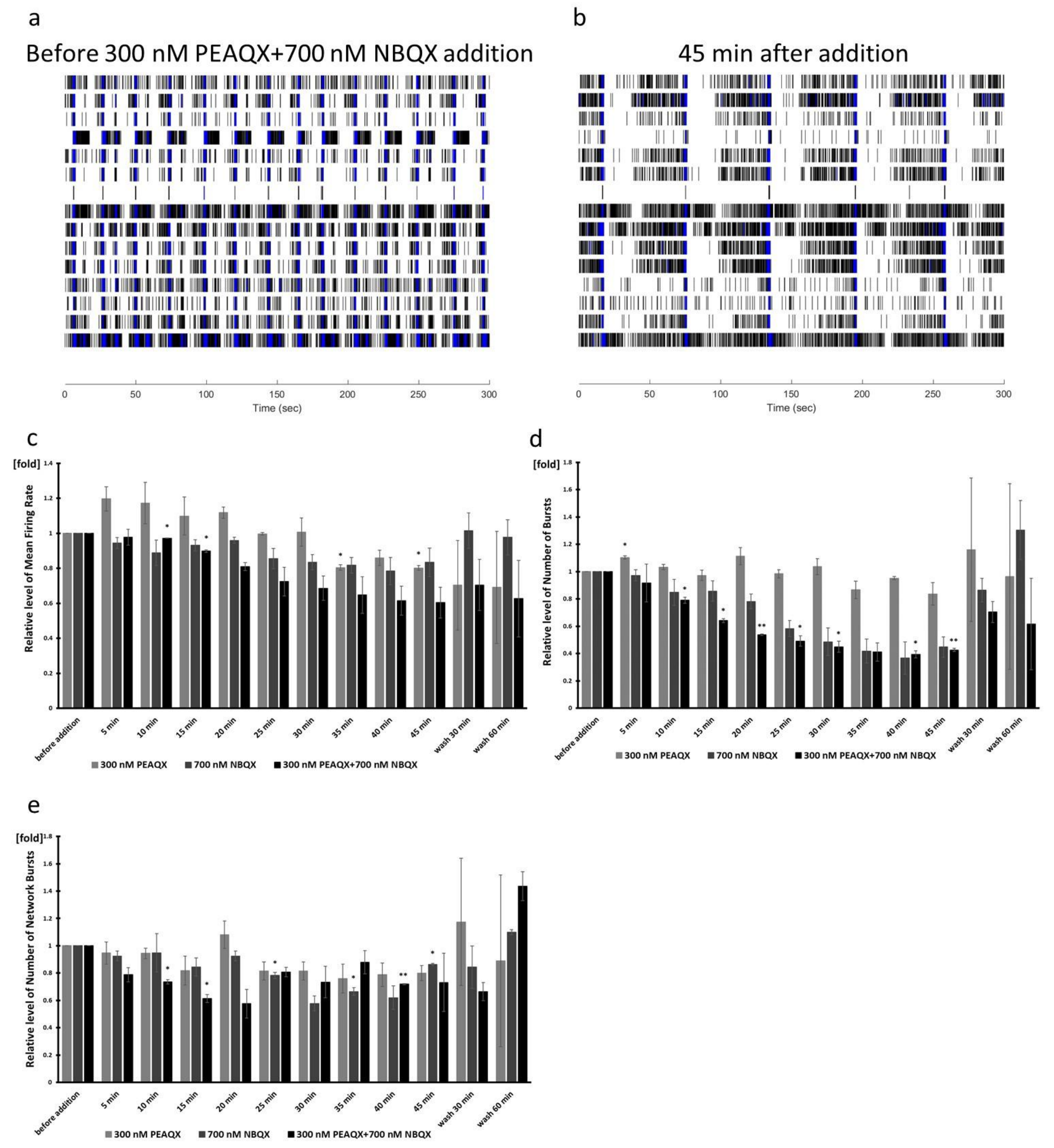

Supplementary FigureS5. Synaptic blockers, NMDA receptor antagonist PEAQX and AMPA receptor antagonist NBQX responsiveness. Raster plots of (a) before 300 nM PEAQX and 700 nM NBQX addition (baseline), (b) 45 min after adding PEAQX and NBQX. (c) Mean Firing Rate, (d) Number of Bursts and (e)Number of Network Bursts normalized to mean firing rate, number of bursts and number of network bursts before drug addition. n=2 wells, *p<0.05, **p<0.01 compared to the value recorded before drug addition.

primer name	sequence (5' to 3')
PRPH Forward	CAAGCAGGAGATGAACGAGTCC
PRPH Reverse	TCCAGCTCTCTCAACTGCCTGA
Nav1.7 Forward	CACAATCCCAGCCTCACAGT
Nav1.7 Reverse	CTGAGGAGCTTGACCGGTTTA
BRN3A Forward	GCACACATTCACACAGTGGTAACAG
BRN3A Reverse	AAGCTCAGCAGACATACATGGACAG
TRPV1 Forward	GAGTTTCAGGCAGACACTGGAA
TRPV1 Reverse	CTATCTCGAGCACTTGCCTCTCT
TRPA1 Forward	CTGTGCAGGGCATGAATAATGAG
TRPA1 Reverse	ATTTGTGGTGCACGCAATGA
TRPM8 Forward	TATACAAAGCCTTCAGCACCA
TRPM8 Reverse	AATCTCATCATTGGCTAAGTCC
Nav1.8 Forward	CTGTCGATGTCTCGGCATTC
Nav1.8 Reverse	TGGGCACTTCTGTTCAGACTC
TRKA Forward	CACTAACAGCACATCTGGAGACC
TRKA Reverse	TGAGCACAAGGAGCAGCGTAGA
TRKB Forward	ACAGTCAGCTCAAGCCAGACAC
TRKB Reverse	GTCCTGCTCAGGACAGAGGTTA
TRKC Forward	CCGACACTGTGGTCATTGGCAT
TRKC Reverse	CAGTTCTCGCTTCAGCACGATG
Piezo2 Forward	ATGGCCTCAGAAGTGGTGTG
Piezo2 Reverse	ATGTCCTTGCATCGTCGTTTT
H1R Forward	CAGAGGATCAGATGTTAGGTGATAGC
H1R Reverse	AGCGGAGCCTCTTCCAAGTAA
P2X3 Forward	GCGTTTCTGAGAAAGCAGCGTG
P2X3 Reverse	CGGATGCCAAAAGCCTTCAGGA
MrgprX1 Forward	CTGGATTTCAAACTGGATTTGAGGA
MrgprX1 Reverse	ACCCTAGTCTGGTGACCCTGGA
CGRP Forward	CCCAGAAGAGAGCCTGTGACA
CGRP Reverse	CTTCACCACACCCCCTGATC
TAC1 Forward	GACAGCGACCAGATCAAGGAGGAA
TAC1 Reverse	GCATTGCACTCCTTTCATAAGCCA
Actin-β Forward	TGAAGTGTGACGTGGACATC
Actin-β Reverse	GGAGGAGCAATGATCTTGAT

Supplementary Table1. List of primers used in real-time PCR.
